# Bisphenol-A and Nonylphenol Induce Apoptosis in Reproductive Tract Cancer Cell Lines by the Activation of ADAM17

**DOI:** 10.3390/ijms19082238

**Published:** 2018-07-31

**Authors:** Paulina Urriola-Muñoz, Raúl Lagos-Cabré, Daniel Patiño-García, Juan G. Reyes, Ricardo D. Moreno

**Affiliations:** 1 Instituto de Química, Pontificia Universidad Católica de Valparaíso, Valparaíso 2340000, Chile; paulina.urriola@gmail.com (P.U.-M.); juan.reyes@pucv.cl (J.G.R.); 2 Departamento de Ciencias Fisiológicas, Facultad de Ciencias Biológicas, Pontificia Universidad Católica de Chile, Alameda 340, Santiago 7820436, Chile; rclagos@uc.cl (R.L.-C.); dafpaga@gmail.com (D.P.-G.)

**Keywords:** apoptosis, endocrine-disruptor chemicals, ADAM17, cancer cell lines

## Abstract

Endocrine-disruptor chemicals (EDCs), such as bisphenol A (BPA) and nonylphenol (NP), have been widely studied due to their negative effects on human and wildlife reproduction. Exposure to BPA or NP is related to cell death, hormonal deregulation, and cancer onset. Our previous studies showed that both compounds induce A Disintegrin And Metalloprotease 17 (ADAM17) activation. Here, we show that BPA and NP induce apoptosis in prostate and ovary cancer cell lines, in a process dependent on ADAM17 activation. ADAM17 knockdown completely prevented apoptosis as well as the shedding of ADAM17 substrates. Both compounds were found to induce an increase in intracellular calcium (Ca^2+^) only in Ca^2+^-containing medium, with the NP-treated cells response being more robust than those treated with BPA. Additionally, using a phosphorylated protein microarray, we found that both compounds stimulate common intracellular pathways related to cell growth, differentiation, survival, and apoptosis. These results suggest that BPA and NP could induce apoptosis through ADAM17 by activating different intracellular signaling pathways that may converge in different cellular responses, one of which is apoptosis. These results confirm the capacity of these compounds to induce cell apoptosis in cancer cell lines and uncover ADAM17 as a key regulator of this process in response to EDCs.

## 1. Introduction

Endocrine-disruptor chemicals (EDCs) is the given name of a wide variety of compounds that disrupt the internal hormonal balance in humans and wildlife animals, leading to transient and/or permanent negative effects [1,2,3,4,5]. Bisphenol A (BPA) and nonylphenol (NP) are two of the most known EDCs used in the plastic and detergent industry [2,6], and these can be found in human biological samples (serum, breast milk, urine, fetal blood, and umbilical cord blood) [2,7]. NP is a degradation product of alkylphenol ethoxylates, which are used in the plastic industry for manufacturing dentistry, food packaging, textiles, pesticides, detergents, paints, and cosmetics [2,7]. On the other hand, the monomer BPA is one of the principal components of polycarbonate plastics and epoxy resins used worldwide [2,7]. BPA contamination occurs due to its leeching from plastics and resins used in the alimentary industry, and eventually reaching food and liquids consumed by humans and animals [2,8]. 

Several studies in animal models have shown adverse effects of BPA and NP in the male reproductive tract, such as reduced testis size, hormonal deregulation, germ cell apoptosis, and decrease in spermatozoa production [1,3,4,9,10,11,12]. Interestingly, EDCs have also been related to a predisposition to several types of cancer [2,5,13,14,15,16]. In this context, it has been shown that low levels of BPA increase the risk of hormone-dependent cancer in human prostate stem-like cells by measuring carcinogenesis markers in BPA-treated samples [5]. Similar effects were observed in mice, where BPA exposure during neonatal period increased prostatic intraepithelial neoplasia incidence in adulthood [17]. Low levels of BPA and NP have also been related to increased migration of BG-1, SKOV3, and A2780 ovarian cancer cells [18,19,20]. Interestingly, BPA treatment upregulates matrix metalloproteases and apoptosis-related genes in the ovarian cancer cell line SKOV3 [20]. This information only adds to the wide range of negative effects of these compounds.

Previously, we have shown that BPA and NP induce the release of Tumor Necrosis Factor α (TNF), Transforming Growth Factor α (TGFα), and Heparin-binding EGF-like Growth Factor (HB-EGF) from plasma membrane by A Disintegrin And Metalloprotease 17 (DAM17) [21,22,23], and that BPA and NP induce apoptosis in male germ cells by activating p38 MAPK and ADAM17 [23,24,25]. ADAM17 is a transmembrane metalloprotease involved in the release of several substrates from the cell surface to the extracellular medium, activating or inhibiting signaling pathways involved in a myriad of cellular and physiological processes [26,27]. Interestingly, a pharmacological blocker of ADAM17 is able to prevent the BPA- and NP-induced apoptosis of germ cells, suggesting it as a putative molecular target [23]. In addition, other studies have shown that in vitro NP induces the apoptosis of neurons and thymocytes [28,29] in a mechanism that depends on the increased intracellular calcium (Ca^2+^) concentration ([Ca^2+^]_i_) induced by NP [30]. Interestingly, both compounds have been shown to modulate in vitro intracellular Ca^2+^ levels in different cell types [31,32,33,34,35], suggesting that it is possible to hypothesize a mechanistic relationship between perturbed Ca^2+^ levels, ADAM17 activation, and cell apoptosis.

In this work we show that BPA and NP differentially induce apoptosis in cancer cell lines in a mechanism dependent upon ADAM17 and extracellular Ca^2+^, and we provide insights of possible mechanisms involved in the activation of ADAM17 by BPA and NP.

## 2. Results 

**Figure 1:**
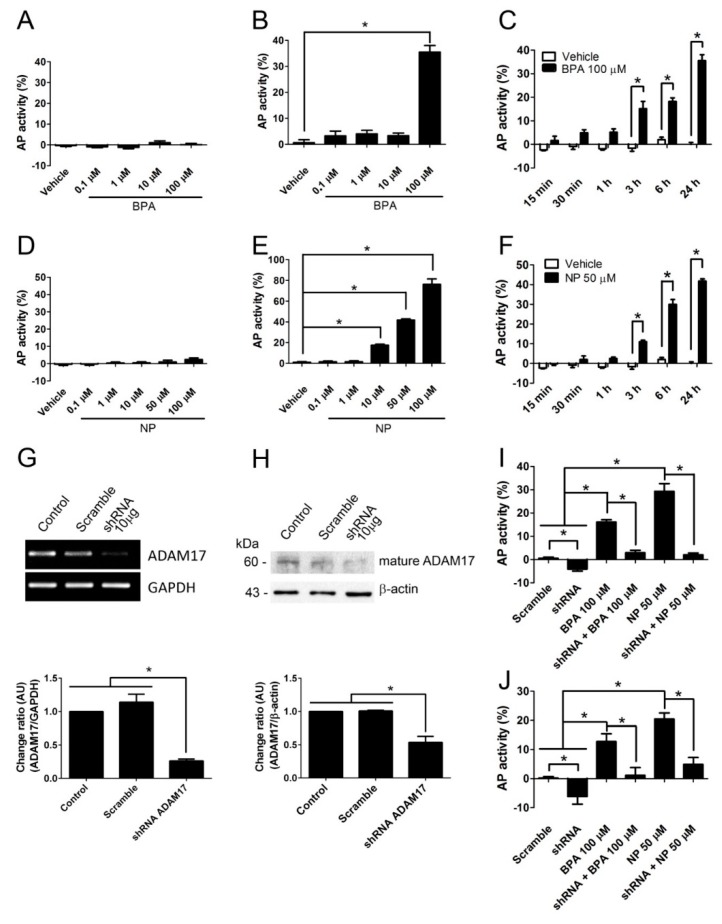
A Disintegrin And Metalloprotease 17 (ADAM17) sheddase activity is induced by bisphenol A (BPA) and nonylphenol (NP) in LNCaP cells transfected with alkaline phosphatase (AP)-neuregulin 1 (NGR1) vector. LNCaP cells show no detectable AP activity in culture medium when incubated with BPA (**A**) or NP (**D**) when they are not transfected with (AP)-NRG1 vector. LNCaP cells transfected with (AP)-NGR1 vector show a significant increase in culture medium when they are incubated for 24 h in the presence of 100 µM BPA (**B**) or 10–100 µM NP (**E**). Time course of AP activity release of transfected LNCaP cells with (AP)-NRG1 vector using 100 µM BPA (**C**) or 50 µM NP (**F**). Transfection of LNCaP cells with 10 µg shRNA induces a robust decrease in the levels of mRNA (**G**) and protein (**H**) of ADAM17. Silencing of ADAM17 with 10 µg shRNA in transfected LNCaP cells with (AP)-NRG1 vector (**I**) or (AP)-Tumor Necrosis Factor α (TNF) vector (**J**) reduces the basal levels of AP shedding and completely prevents the effect of BPA and NP. Vehicle: ethanol, mean ± SEM, * *p* < 0.05, *n* = 3.

First, we determined that the LNCaP cell line expresses ADAM17 (Figure S1). Then, LNCaP cells were transfected with a plasmid containing a substrate of ADAM17, neuregulin 1 (NRG1), coupled to alkaline phosphatase (AP) ((AP)-NRG1), which allowed us to detect its shedding by measuring the AP activity in the cell medium [36]. There was no detection of AP activity in the culture medium of non-transfected cells when they were incubated with different concentrations of BPA or NP for 24 h (Figure 1A,D). However, a dramatic increase in the activity was detected when transfected cells were incubated with 100 μM BPA, or with 10, 50, or 100 μM NP (Figure 1B,E). The kinetics of (AP)-NRG1 shedding using 100 μM BPA or 50 μM NP showed that after only 3 h AP activity was detected in the culture medium and steadily increased up to 24 h of incubation (Figure 1C,F), which suggests a rapid response to these compounds. AP activity in the culture medium was not due to toxicity by plasma membrane permeabilization, since incubation with the concentrations of BPA and NP for 24 h used in these experiments did not increase cell trypan blue uptake (Figure S2A,B) and cell morphology was not affected (Figure S2C).

Next, we evaluated if the presence of ADAM17 was necessary to induce release of (AP)-NRG1 after BPA or NP exposure. To this end, we knocked down ADAM17 using a specific shRNA against this metalloprotease (Figure 1G,H), resulting in about 70% reduction of the mRNA and 50% at the protein ADAM17 levels using the antisense, but not scrambled shRNA. As shown before, treatment with 100 μM BPA or 50 μM NP stimulates a robust release of (AP)-NRG1 as compared with treatment with scrambled shRNA (Figure 1I). The knockdown of ADAM17 totally prevented the shedding of (AP)-NRG1 after treatment with 100 μM BPA or 50 μM NP. Interestingly, levels of (AP)-NRG1 in the culture medium were reduced in cells treated with shRNA as compared to scrambled shRNA, suggesting that in these cells the basal release of this protein depends on ADAM17.

To further confirm these results, we transfected LNCaP cell lines with another ADAM17 substrate, TNF coupled to AP, (AP)-TNF. Results showed that 100 µM BPA or 50 µM NP strongly stimulated the release of (AP)-TNF and that the knockdown of ADAM17 prevented the shedding of this substrate to basal levels (Figure 1J). As showed before, shRNA treatment reduced levels of (AP)-TNF as compared to those treated with scrambled RNA, suggesting that the basal release of TNF as well as NRG1 depends upon ADAM17.

Taken together, these results strongly suggest that in vitro BPA and NP induce ADAM17 activity in LNCaP cell lines.

### 2.1. BPA and NP Induced Apoptosis in LNCaP Requires ADAM17

**Figure 2:**
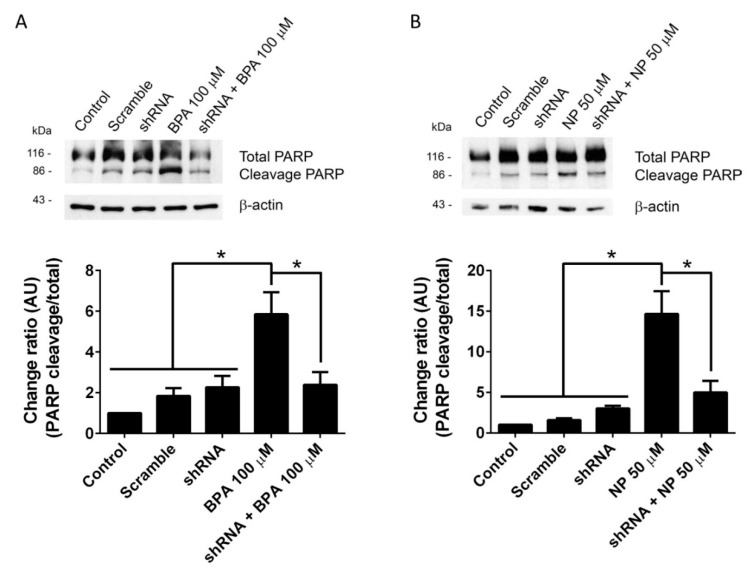
Silencing of ADAM17 prevents poly (ADP-ribose) polymerase (PARP) cleavage induced by BPA or NP in LNCaP cells. Treatment with 100 µM BPA (**A**) or 50 µM NP (**B**) for 6 h induces a significant increase in the cleaved form (86 kDa) of PARP detected by Western blot. Silencing of ADAM17 with 10 µg shRNA prevents the increase of the 86 kDa form in LNCaP cells treated with BPA (**A**) or NP (**B**). Mean ± SEM, * *p* < 0.05, *n* = 3.

Apoptosis is a type of cell death characterized by the activation of a group of cysteine-proteases named caspases, among which caspase-3 is the major executioner of this process and proteolytically inactivates different intracellular proteins, leading to cell dismantlement [37,38]. Poly (ADP-ribose) polymerase (PARP) is one of the caspase-3 substrates belonging to a family of proteins involved in a number of cellular processes such as DNA repair and genomic stability, and its proteolysis is used as a measure of caspase-3 activation [39]. Related to this, from 15 min of 100 μM BPA treatment or from 3 h of 50 μM NP treatment, a significant increase in the number of active caspase-3-positive cells was observed in LNCaP (Figure S3). Using PARP cleavage as a criterion of caspase-3 activation, we determined that treatment with 100 μM BPA and 50 μM NP, which are concentrations that stimulate the shedding of ADAM17 substrates, induces a significant increase in cleaved PARP levels (Figure 2A,B). When ADAM17 was knocked down by shRNA, the increase of cleaved PARP induced by BPA and NP was decreased significantly and reached basal levels, suggesting that BPA and NP activate apoptotic pathways in an ADAM17-dependent manner.

**Figure 3:**
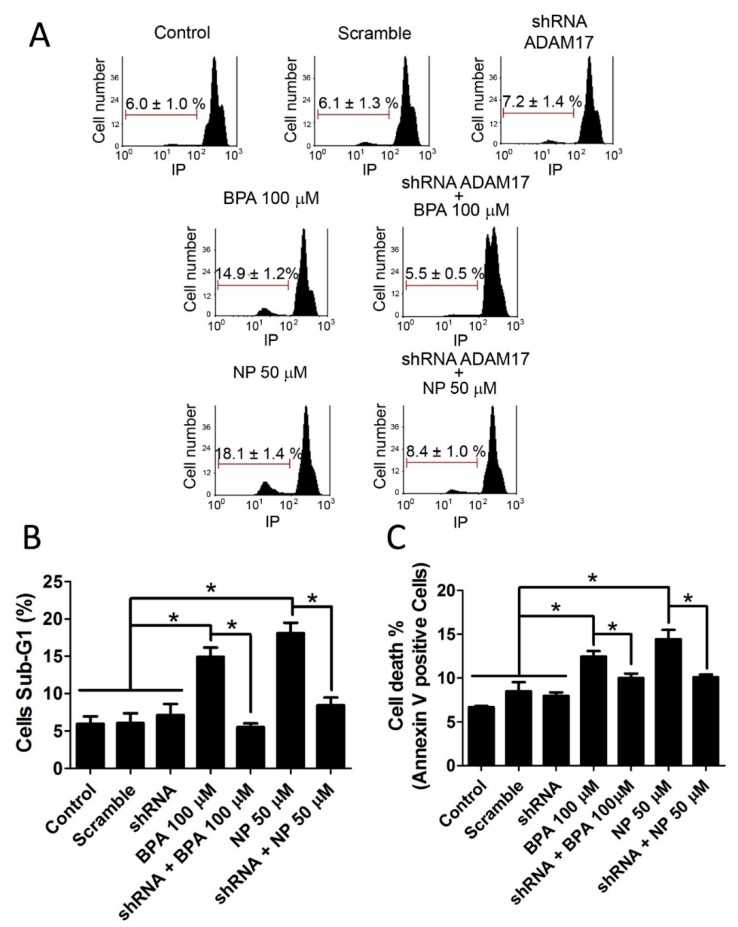
Silencing of ADAM17 prevents an increase in the sub-G1 population and Annexin-V-positive cells induced by BPA or NP in LNCaP cells. (**A**) Sub-G1 population analysis in LNCaP cells after 6 h of exposure to 100 µM BPA or 50 µM NP. Marker in each panel indicates sub-G1 cell population. ADAM17 knocked down with 10 µg shRNA prevents the increase in the percentage of sub-G1 cell population (**B**) or Annexin-V-positive cells (**C**) induced by BPA or NP. Mean ± SEM, * *p* < 0.05, *n* = 3.

Apoptosis was also evaluated by the sub-G1 population, which represents cells with fragmented and condensed DNA unable to fully incorporate PI. Results show that BPA and NP significantly increase the sub-G1 population, which was prevented by knocking down ADAM17 (Figure 3A,B). In addition, apoptosis was further evaluated using Annexin-V, that binds externally to phosphatidylserine, flipping to the outer plasma membrane early after apoptotic stimuli [37]. The results showed that the percentage of Annexin-V-positive cells increased after incubation with 100 µM BPA or 50 µM NP, and this was prevented by knocking down ADAM17 (Figure 3B). 

**Figure 4:**
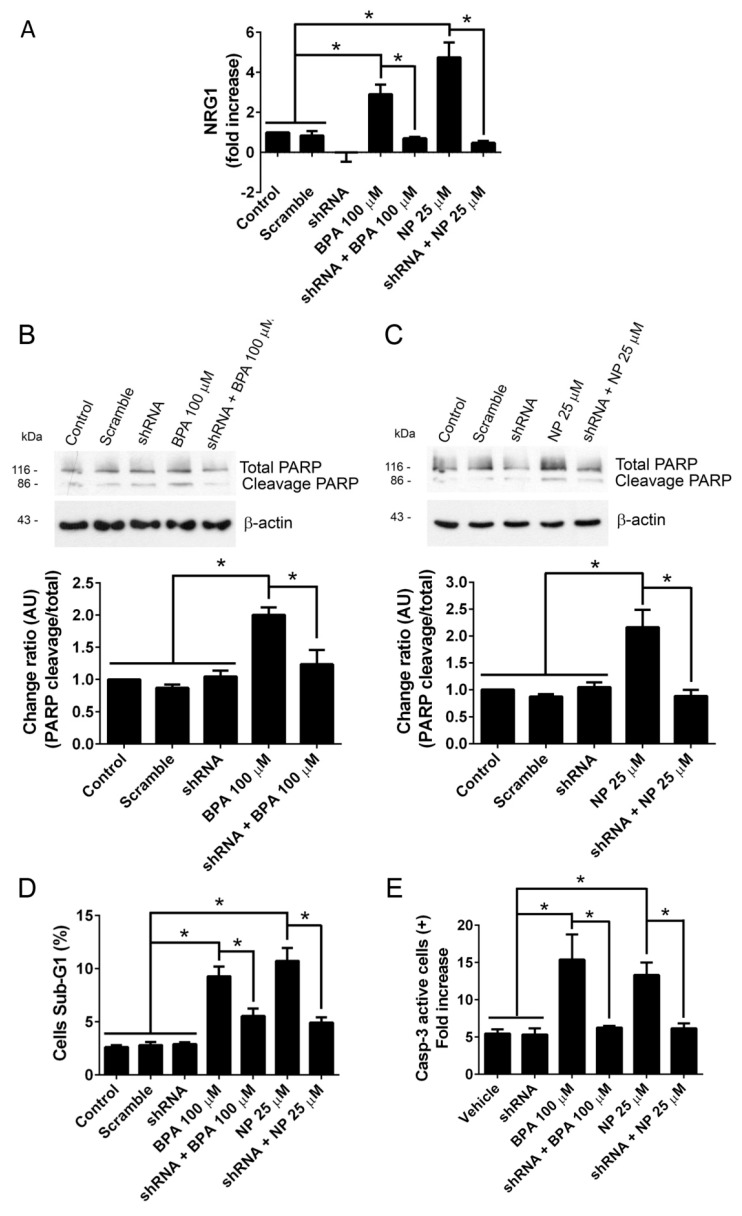
Silencing of ADAM17 prevents NRG1 shedding and apoptosis induced by BPA or NP in A2780 cells. (**A**) Shedding of NRG1 significantly increases in A2780 cells transfected with (AP)-NRG1 vector when they are incubated for 2 h in the presence of 100 µM BPA or 25 µM NP. This effect is prevented when ADAM17 is silenced by the use of 10 µg shRNA. Treatment with 100 µM BPA (**B**) or 25 µM NP (**C**) for 2 h induces a significant increase in the cleaved form (86 kDa) of PARP detected by Western blot, which was prevented when ADAM17 was silenced with 10 µg shRNA. Treatment with 100 µM BPA or 25 µM NP for 2 h induces a significant increase in the sub-G1 population (**D**) and cleaved caspase-3-positive cells of A2780 cells line (**E**), which were prevented when ADAM17 was knocked down with 10 µg shRNA. Mean ± SEM, * *p* < 0.05, *n* = 3.

To make sure that our findings were not related to a specific cell line, we used the A2780 cell line, which is derived from human ovarian carcinoma [40]. This cell line express ADAM17 and it can be also knocked down using shRNA (Figure S4). A transiently transfected A2780 cell line with (AP)-NRG1 showed increased levels of AP activity in cell culture medium (Figure 4A) and cleaved PARP when incubated with 100 μM BPA or 25 μM NP (Figure 4B,C). Apoptosis was confirmed by the increase in the sub-G1 population (Figure 4D) and the number of active caspase-3-positive cells (Figure 4E) observed. All these effects induced by BPA or NP were completely prevented when knocking down ADAM17 (Figure 4). Neither BPA nor NP permeabilized the A2780 cell lines, as evaluated by trypan blue intake (Figure S2D,E). They also did not affect cell morphology (Figure S2F). As a whole, these results strongly suggest that in vitro BPA and NP induce ADAM17-dependent apoptosis in at least two different cancer cell lines, LNCaP and A2780.

### 2.2. Pathways Activated by BPA and NP

**Figure 5:**
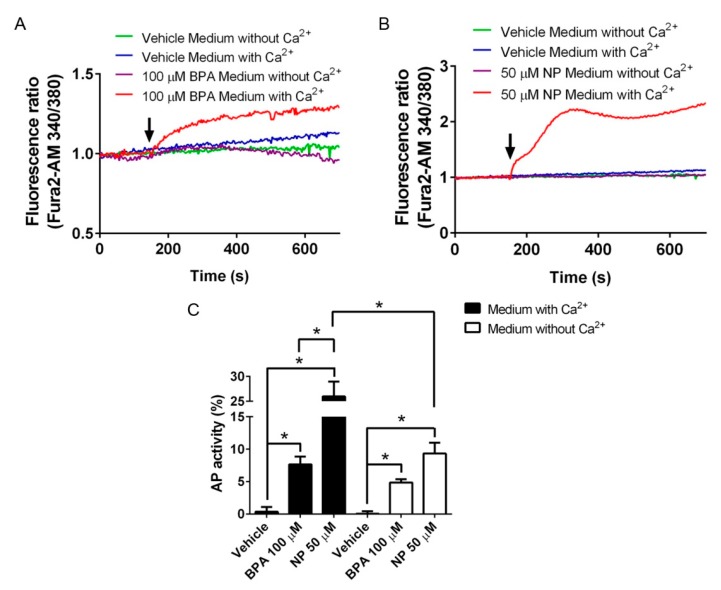
Participation of calcium (Ca^2+^) on the sheddase activity induced by BPA and NP in LNCaP cells. Intracellular Ca^2+^ levels after 100 µM BPA (**A**) or 50 µM NP (**B**) treatment in a medium with or without Ca^2+^. (**C**) Shedding of NRG1 in (AP)-NRG1 transfected LNCaP cells, when they are incubated for 3 h in the presence of 100 µM BPA or 50 µM NP, in a medium with (black bars) or without (white bars) Ca^2+^. Mean ± SEM, * *p* < 0.05, *n* = 5.

It is known that BPA and NP induce an increase in the Ca^2+^]_i_ in different cell types [22,23]. Specifically, 100 μM BPA or 50 μM NP induced a fast and sustained increase in [Ca^2+^]_i_, which was not observed in Ca^2+^-free medium or after vehicle treatment (Figure 5A,B). LNCaP cells transiently transfected with (AP)-NRG1 showed a robust increase in AP activity after incubation with 100 µM BPA or 50 µM NP in a Ca^2+^-free medium (Figure 5C, white bars). The shedding of (AP)-NRG1 after 100 µM BPA was not significantly affected by extracellular Ca^2+^ (Figure 5C). However, the shedding of (AP)-NRG induced by NP was potentiated in Ca^2+^-containing medium (Figure 5C, compare white and black bars), suggesting that a greater effect of NP relies on extracellular Ca^2+^. These results show that in the absence of extracellular Ca^2+^, BPA and NP induce similar ADAM17 sheddase activity, but only NP-induced activity is potentiated by extracellular Ca^2+^, probably by increasing [Ca^2+^]_i_.

**Figure 6:**
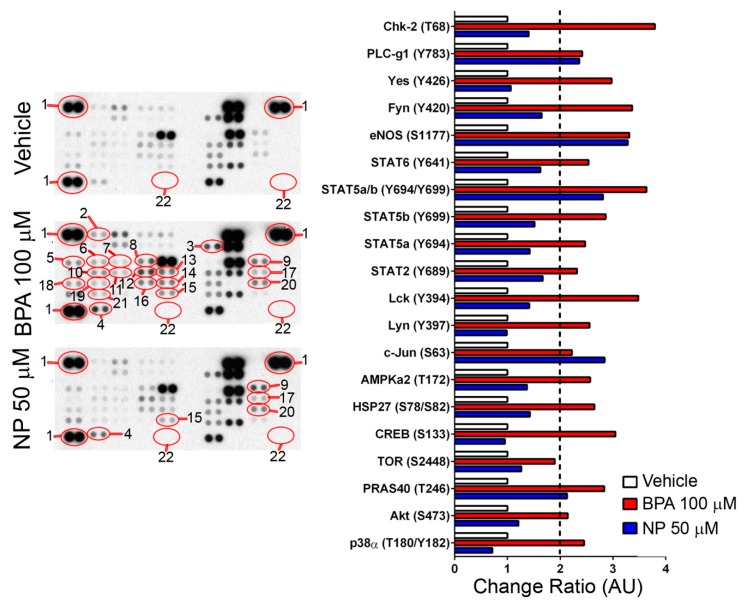
BPA or NP induces an increase in the phosphorylation of protein kinases analyzed by a human phospho-kinase antibody array. LNCaP treated with 100 µM BPA or 50 µM NP for 15 min shows an increase of several protein kinases. The graph shows the quantification of the pixels density shown in the left panel. The kinases selected were those whose change ratio was at least two times higher compared to those observed in the vehicle treatment (ethanol). (1) Reference spot, (2) p38α (T180/Y182), (3) Akt (S473), (4) PRAS40 (T246), (5) TOR (S2448), (6) CREB (S133), (7) HSP27 (S78/S82), (8) AMPKa2 (T172), (9) c-Jun (S63), (10) Lyn (Y397), (11) Lck (Y394), (12) STAT2 (Y689), (13) STAT5a (Y694), (14) STAT5b (Y699), (15) STAT5a/b (Y694/Y699), (16) STAT6 (Y641), (17) eNOS (S1177), (18) Fyn (Y420), (19) Yes (Y426), (20) PLC-γ1 (Y783), (21) Chk-2 (T68), and (22) negative control (PBS).

**Figure 7:**
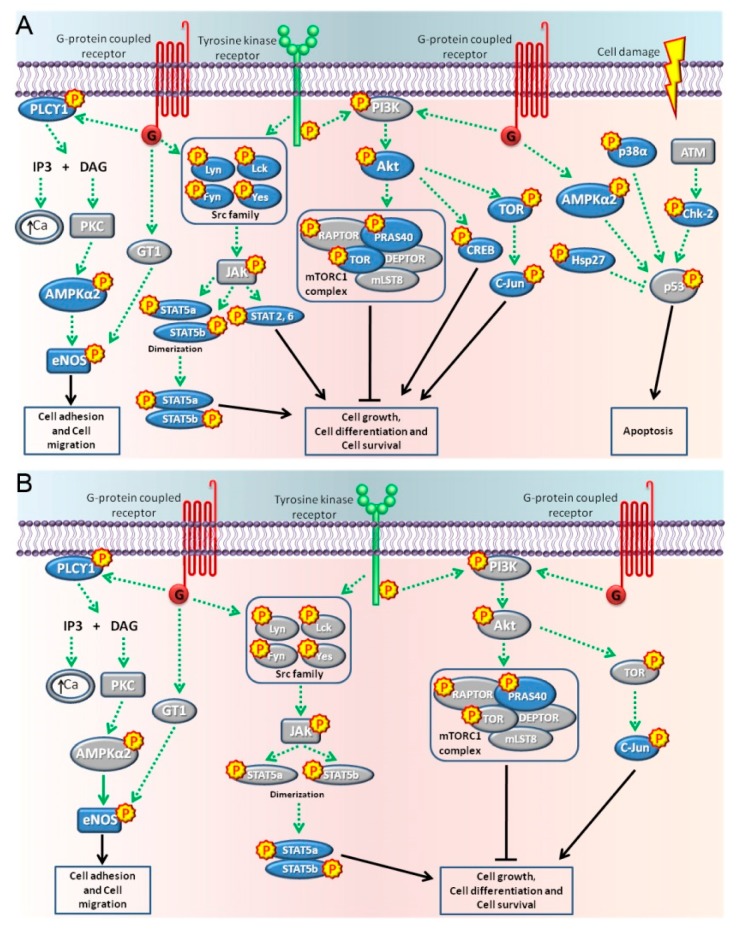
Diagram of possible signaling pathways induced by a 15-min treatment with BPA (**A**) or NP (**B**) in LNCaP cells. BPA mainly activated G protein-coupled membrane receptors and tyrosine kinase receptors or cellular damage pathways (**A**), while NP mainly activated G protein-coupled membrane receptors and tyrosine kinase receptor pathways (**B**). The phosphorylated proteins participating in pathways of: cell adhesion and cell migration, cell growth, cell differentiation and cell survival, and apoptosis. In blue are shown proteins that increase their levels of phosphorylation as a result of the treatment, while in gray are shown proteins that were analyzed did not show a significantly increase in phosphorylation.

In an attempt to elucidate the intracellular signaling pathways elicited by BPA and NP in LNCaP cells, we took advantage of a human phospho-kinase antibody array that detects changes in the phosphorylation state of 43 signaling-related phosphoproteins (Table S1). We found that after a 15-min treatment with 100 μM BPA or 50 μM NP, 20 and five protein kinases out of these 43 phosphoproteins detected by the antibody array (Table S1), respectively, showed at least 2-fold increased protein levels, as compared with the vehicle (Figure 6). Interestingly, all five kinases activated by NP were also activated by BPA, and corresponded to PLC-γ1, eNOS, STAT5a/b, PRAS40, and c-Jun (Figure 6 and Figure 7).

It was observed that BPA activated pathways related to the G-protein coupled receptors, tyrosine kinase receptors, and pathways activated by cell damage (Figure 7A). The PLCγ1, AMPKα2, and eNOS proteins participated in the GPCRs pathways inducing cell adhesion and cell migration [41,42,43,44,45,46,47] (Figure 7A). The kinases Akt, Lyn, Lck, Yes, Fyn PRAS40, TOR, CREB, c-Jun, STAT5a, STAT5b, and the dimers STATa/b, 2 and 6 were activated mostly by tyrosine kinase receptors participating in cell growth, cell differentiation, and cell survival pathways [48,49,50,51,52,53,54,55,56,57,58,59] (Figure 7A). Finally, the kinases p38α, Hsp27, and Chk-2 were activated by cell damage in the form of heat stress and treatments with anticancer drugs that induce apoptosis [60,61,62,63] (Figure 7A). On the other hand, a treatment with NP for 15 min mainly activated the cell adhesion and cell migration pathways due to the phosphorylation of PLCγ1 and eNOS, probably by GPCRs mediated pathways, and activated the tyrosine kinase receptors due to the phosphorylation of STAT5a/b, PRAS40, and c-jun participating in cell growth, cell differentiation, and cell survival pathways [41,42,43,47,49,50,54,55] (Figure 6 and Figure 7B). Taken together, these results suggest that BPA and NP elicit different intracellular signaling pathways that could lead to ADAM17 sheddase activation. 

## 3. Discussion

Previous work in cultured cells have shown that BPA and NP induce apoptosis in different cell types [23,25,28,29,34]. On the contrary, low concentrations (nM or pM) of BPA and NP induce cell proliferation in transformed and non-transformed cells [64,65,66]. Here, we report that BPA and NP, at concentrations similar to those found in the urine of humans exposed to high concentrations of these EDCs, such as plastic factory workers, who are exposed up to 500 times higher levels of these compounds than the general population [67], induce apoptosis in prostate (LNCaP) and ovary (A2780) cancer-derived human cell lines. Our experimental strategy included four different approaches to establish that, in vitro, concentrations of 100 μM BPA or 50 μM NP effectively induce apoptosis in two different cancer cell lines. Our previous findings have suggested that, in vivo, BPA and NP induce germ cell apoptosis by a mechanism that involves the activation of the metalloprotease ADAM17 [23]. Here, we show that, in vitro, BPA- and NP-induced apoptosis could be prevented by knocking down ADAM17, suggesting that this enzyme is important in the mechanism elicited by these compounds to induce cell death. Interestingly, the concentrations that induce cell death are the same as those that stimulate the release of ADAM17 substrates (TNF and NRG1), suggesting that the shedding of ADAM17 substrates might be connected to the induction of apoptosis. In this way, it is possible that the mechanisms involved in the shedding of ADAM17 substrates, or the substrates released after the stimulus, could participate in the elicitation of apoptosis in an auto/paracrine pathway.

It is possible that substrates such as TNF or FAS ligand (FASL) could bind to its cognate receptor in an auto/paracrine way and stimulate apoptosis only in a particular group of cells expressing these receptors or in a specific stage of the cell cycle. Interestingly, it has been documented that nonylphenol-induced apoptosis in a human embryonic stem cell and two human bronchial epithelial cell lines is related to the FASL/FAS system [68]. This hypothesis could explain the fact that no more than 20% of the exposed cells undergo apoptosis.

It has been reported that BPA at low concentrations causes an increase in epidermal growth factor receptor (EGFR) activation and the downstream ERK pathway in inflammatory breast cancer cells, which is associated with increased cell proliferation [69]. In the same way, BPA and NP stimulate the release of EGFR ligands, such as TGFα or HB-EGF, by activating either ADAM17 or ancillary proteins such as iRhom1 and/or iRhom2 [22,70]. The activation of EGFR by BPA was also observed in LNCaP cells, which leads to the activation of the main cellular regulator p53 [71]. Here, we show that BPA or NP activate a set of phosphoproteins participating in different intracellular pathways involved in cell death, proliferation, and differentiation. However, interestingly, p53 was one of the proteins that was not affected by either treatment. The activation of p53 appears to be a late effect of BPA treatment, which is observed after 24 h [71]. In our experiments, BPA and NP induce apoptosis at 6 h, which could explain the absence of this protein in our phosphoprotein microarray. BPA activates more phosphoproteins than NP, suggesting that the former has a wider range of effects in different cell contexts than the latter. Interestingly, we found that all of the phosphoproteins that increased protein levels after NP treatment were also increased after BPA treatment, suggesting that both compounds share some signaling pathways. Since both compounds activate the shedding of ADAM17 substrates, we suggest that it is possible that two of the common activated phosphoproteins—c-Jun and Stat5a/b—could explain these effects. In fact, it has been observed that the modulation of c-Jun or Stat5a activation also modulates TNF shedding elicited by BPA and NP in cancer cell lines [72,73,74]. However, it remains to be investigated whether the activation of the phosphoproteins observed in the present study is due to a direct activation of BPA and NP or whether this activation is achieved indirectly, by the shedding and auto/paracrine activation of cognate receptors, which seems to be the case of BPA [69].

Finally, we confirmed previous results showing that BPA and NP induce a rise in [Ca^2+^]_i_, which depends upon the presence of extracellular Ca^2+^. This effect could be related to the different Ca^2+^ channels that are activated by either of these compounds in different cell types [31,32,33,75,76]. Interestingly, the ADAM17 activation induced by NP was higher than that induced by BPA in Ca^2+^-containing medium, but in a Ca^2+^-free medium BPA- and NP-induced ADAM17 sheddase activity was similar, demonstrating that both compounds have a similar basal effect. In this context, it is interesting that we detected that BPA and NP activate PLCγ, which produces the second messengers IP_3_ and DAG, which open intracellular Ca^2+^ stores and subsequently may activate PKC. However, it is possible that under our experimental conditions, activated PLCγ reached levels below the activation threshold to produce enough IP_3_ in order to open the intracellular Ca^2+^ stores, which could explain the absence of Ca^2+^ signals when cells were treated in a Ca^2+^-free medium.

The present results extend our previous observations that BPA- and NP-induced germ cell apoptosis requires ADAM17 activation, suggesting a general mechanism of cell toxicity for these EDCs that may also include extracellular Ca^2+^ as a potentiator of the effect.

## 4. Materials and Methods

### 4.1. Chemicals and Antibodies

Bisphenol A [2,2-bis (4-hydroxyphenyl) propane] (BPA) (239658) and nonylphenol (NP) (442873) were obtained from Sigma (St. Louis, MO, USA). LipofectAMINE 2000 (11668-027), p-nitrophenyl phosphate (p-NPP) (002212), substrate of alkaline phosphatase, TRIzol-Reagent (15596026), FURA2-AM (F1221), and propidium iodide (P1304MP) were obtained from Invitrogen (Carlsbad, CA, USA). BB-94 (Batimastat) (196440) was obtained from Calbiochem (San Diego, CA, USA). ADAM17 antibody (ab39163) was purchased from Abcam (Cambridge, MA, USA). PARP-1/2 antibody (sc-7150) was purchased from Santa Cruz Biotechnology (Santa Cruz, CA, USA). Cleaved Caspase-3 (Asp175) antibody (#9661) was acquired from Cell Signaling (Danvers, MA, USA). β-actin (AC-15) and anti-rabbit IgG-FITC (F0382) antibodies were purchased from Sigma (St. Louis, MO, USA). Peroxidase anti-mouse IgG (5220-0286) and peroxidase anti-rabbit IgG (5220-0283) antibodies were obtained from KPL (Gaithersburg, MD, USA). Human phospho-kinase antibody array (Catalog #ARY003B) was obtained from R&D Systems (Minneapolis, MN, USA). Western Lightning Chemiluminescense Reagent Plus kit (NEL104001EA) was obtained from PerkinElmer Inc. (Waltham, MA, USA).

### 4.2. Cell Transfection and Generation of a Stable Cell Line

LNCaP was kindly donated by Dr. Alejandro Godoy from Pontificia Universidad Católica de Chile (Santiago, Chile), and A2780 was kindly donated by Dr. Gareth Owen from Pontificia Universidad Católica de Chile (Santiago, Chile). Both cell lines were transfected with the plasmid (AP)-NRG1 or (AP)-TNF [36,77], kindly donated by Dr. Carl Blobel (Hospital for Special Surgery, New York, NY, USA), or with the shRNA against the ADAM17 mRNA (KH00343N, Qiagen Sicences, MD, USA), which was purchased from Qiagen (Hilden, Germany). Briefly, LNCaP and A2780 cells, after being seeded to 80% confluence for 12 h, were washed and cultured in DMEM-F12 medium (12400-024, Gibco, Life Technologies, Carlsbad, CA, USA) deprived of serum, antibiotic, and antimycotic for 1 h. Following that, the cells were cultured for 6 h with a complex DNA-LipofectAMINE 2000, and finally the cells were cultured for 24 h in DMEM-F12 medium with 10% Fetal Bovine Serum (FBS) (04-127-1A, Biological Industries, Beit Haemek, Israel) and 10% antibiotic and antimycotic (15240-062, Gibco, Life Technologies, Carlsbad, CA, USA). The transfection was evaluated by the presence of NRG1 or the decrease of ADAM17 mRNA levels by RT-PCR.

The generation of LNCaP cell lines that stably expressed (AP)-NRG1 was obtained by the incubation of the transfected cells with 200 µg/mL of antibiotic zeocin (R25001, Invitrogen, Carlsbad, CA, USA). 

### 4.3. Alkaline Phosphatase Activity Measurement

LNCaP cells stably transfected with (AP)-NRG1 were washed once in PBS and incubated for 1 h in DMEM-F12 (nonstimulated conditioned medium) and then in DMEM-F12 containing BPA or NP at different concentrations and for different times (stimulated conditioned medium). Conditioned media were collected and incubated for 1 h with p-NPP (p-nitrophenyl phosphate) (4:1), a colorimetric and soluble AP substrate, and then the AP activity was measured by a spectrophotometer at 405 nm. Each experiment was conducted at least three times. For the A2780 cells, the day following transfection, each well was washed once in PBS and incubated for 1 h in DMEM-F12 (nonstimulated conditioned medium) and then in DMEM-F12 containing BPA or NP at different concentrations for 2 h (stimulated conditioned medium). Conditioned media were collected and cells were lysed in PBS containing 1% Triton X-100 (T8787, Sigma, St. Louis, MO, USA) plus a general metalloprotease inhibitor, BB-94 10 µM, and protease inhibitor cocktail (P2714, Sigma, St. Louis, MO, USA) including 2 mM AEBSF [4-(2-Aminoethyl) benzenesulfonylfluoride hydrochloride], 0.3 μM aprotinin, 130 μM bestatin hydrochloride, 14 μME-64, 1 mM EDTA, and 1 μM leupeptin hemisulfatein. Alkaline phosphatase activity measure was performed as previously published [22]. Briefly, conditioned media and cells lysate were incubated for 1 h with p-NPP (p-nitrophenyl phosphate) (1:1 and 0.1:1, respectively), and then the AP activity was measured by a spectrophotometer at 405 nm. All experiments were conducted in triplicates, and the transfection efficiency was measured by the ratio of AP activity in the medium to that of the cell lysate plus the medium. The fold increase in the ratio of AP activity obtained after our treatments is shown relative to the ratio of AP activity in untreated control wells, which represents the baseline of each graph (value 0 in the Y axis in each graph). In this way, all values are normalized by untreated controls and each value in the graphs represents whether the AP activity was above or below this control.

### 4.4. Protein Extraction and Western Blotting

Protein extraction and Western blotting were performed as previously published [23]. Briefly, the homogenization of cells was performed in a buffer containing 1M NaCl, 1mM EDTA, 10 mg/mL PMSF, 1% Triton X-100, and 20mM Tris–HCl pH 7.4, plus a general metalloprotease inhibitor, BB-94 10 µM, and protease inhibitor cocktail (Sigma), and then centrifuged for 10 min at 10,000× *g* at 4 °C. Then, samples were run on a 10% polyacrylamide gel under reducing and denaturing conditions, transferred to nitrocellulose at 400 mA for 1 h, and blocked with 3% (*w*/*v*) non-fat milk, 0.1% Tween in TBS, pH 7.4. Primary antibodies were incubated overnight at 4 °C with the following dilutions: anti-ADAM17 and anti-PARP-1/2 (0.2 µg/mL), or anti-β-actin (0.3 µg/mL). A secondary antibody conjugated with horseradish peroxidase (0.3 µg/mL) (KPL, Gaithersburg, MD, USA) was used. Protein bands were developed using the Western Lightning Chemiluminescense Reagent Plus kit (PerkinElmer Inc, Waltham, MA, USA).

### 4.5. RNA Extraction and RT-PCR

RNA extraction and PCR were performed as previously reported [23]. Briefly, RNA of LNCaP and A2780 cells was extracted with TRIzol-Reagent (Invitrogen, Carlsbad, CA, USA). Then, cDNA was generated from 1 μg RNA using random primers and Reverse Transcriptase (Promega, Madison, WI). PCR was performed using the PCR Master Mix (Promega). Primer sets: ADAM17 forward 5′-GTTGGTGAGCCTGACTCTA-3′ and reverse 5′-CCTCTTGTGGAGACTTGA-3′; NRG1 forward 5′-ATCCACGACTGGGACCAG-3′ and reverse 5′-AAGCTTCTGCCGCTGTTTC-3′; GAPDH forward 5′-TCCACCACCCTGTTGCTGTA-3′ and reverse 5′-ACCACAGTCCATGCCATCAC-3′. PCR products were run in a 1% agarose gel and then stained with SYBR^®^ Green (Invitrogen, Carlsbad, CA, USA). The pixel density of each band was measured and normalized to GAPDH mRNA levels.

### 4.6. Sub-G1 Population Analysis by Flow Cytometry

After treatment, LNCaP and A2780 cells were fixed in 70% ethanol. The cells were then pelleted and washed once with PBS. Then, the pellet was dissolved in a buffer containing 0.1% sodium citrate, 0.3% Triton X-100 (Sigma), 50 mg/mL propidium iodide, and 50 mg/mL RNase A (Invitrogen, Carlsbad, CA, USA). The samples were then analyzed by a flow cytometer (FAScanto, BD Biosciences, Franklin Lakes, NJ, USA). Ten thousand gated events were acquired in each sample. All data were analyzed with software FCS express V4.0 (De Novo Software, Los Angeles, CA, USA).

### 4.7. Annexin-V Assay 

LNCaP cells treated with 100 µM BPA or 50 µM NP for 6 h were washed twice with PBS. After that, they were incubated with 5 µL of FITC-Annexin-V and 5 µL of propidium iodide for 15 min at room temperature in the dark. Then, 400 µL of binding buffer was added and analyzed by flow cytometry (FAScanto, BD Biosciences, Franklin Lakes, NJ, USA) and 10,000 gated events were acquired in each sample. All data were analyzed with software FCS express V4.0 (De Novo Software, Los Angeles).

### 4.8. [Ca^2+^]_i_ Measurements of LNCaP Cells in Suspension 

LNCaP cells were grown on 10-cm plates for 24 h. After that, they were washed with PBS and then incubated with EDTA 5 mM for 15 min at 37 °C. LNCaP cells were resuspended (20 × 10^6^ cells/mL) in Locke medium (NaCl 130 mM, Ca2Cl 2.3 mM, KCl 5.6 mM, Hepes 8.4 mM, Mg2Cl 1 mM, glucose 5.6 mM). The cells in Locke medium were loaded with 5 μM of the Ca^2+^ probe Fura-2 AM by incubation for 30 min at 37 °C, followed by three washes with Locke medium with Ca^2+^ or Locke medium without Ca^2+^ (NaCl 130 mM, KCl 5.6 mM, Hepes 8.4 mM, Mg2Cl 1 mM, glucose 5.6 mM, EGTA 1 mM). The fluorescence measurements were performed by adding a concentrated cell suspension (50 μL) to a temperature-regulated spectrofluorimeter cuvette (3.0 mL, with stirring) containing 2.5 mL of Locke medium with or without Ca^2+^. The [Ca^2+^]_i_ determinations were carried out using a radiometric method described before [78].

### 4.9. Human Phospho-Kinase Array

The assays were performed using LNCaP cells treated with 100 µM BPA or 50 µM NP for 15 min according to the manufacturer’s protocol (R&D Systems, Minneapolis, USA, Cat: ARY003B). Spots obtained were analyzed measuring the pixel density with Adobe Photoshop 7.0 (Adobe System Incorporated, San Jose, CA, USA). A protein was considered to be upregulated when its spot density was at least 2-fold higher than the corresponding spot observed in the vehicle treatment.

### 4.10. Immunohistochemistry and Immunofluorescence

Active caspase-3 was detected in LNCaP cells seeded on 12-mm cover glasses at 80% confluence and fixed in paraformaldehyde 4%. Samples were first treated with 3% H_2_O_2_ for 10 min, and then incubated with a standard protein block system (Ultra V block, Thermo Scientific, Fremont, CA, USA) for 10 min. Primary rabbit polyclonal antibody against cleaved caspase-3 (Asp175) (#9661, Cell Signaling, Danvers, MA, USA) was applied at a dilution of 1:250 in 3% BSA in TBS containing 0.1% Tween-20; samples were then incubated overnight at 4 °C. Biotinylated secondary antibody, and peroxidase-conjugated streptavidin were applied for 10 min (Thermo Scientific, Fremont, CA, USA). Cover glasses were washed three times for 5 min in Tris–HCl buffer, pH 7.6, with 0.3 M NaCl and 0.1% Tween-20. For final staining, DAB (3,3-diaminobenzidine tetrahydrochloride) plus substrate and chromogen (Thermo Scientific, Fremont, CA, USA) were applied for 1 min. After washing with distilled water, samples were counter-stained with hematoxylin and observed under a phase contrast microscope (Optiphot-2, Nikon, Tokyo, Japan) and photographed with a digital camera (CoolPix 4500, Nikon, Tokyo, Japan). For immunofluorescence, a similar protocol was applied. Briefly, after fixation, permeabilization, and blocking with BSA 3%, cells were stained with anti-ADAM17 antibody for 1 h at room temperature. Then, anti-rabbit FITC at 4 μg/mL diluted in 3% BSA was applied and incubated for another 1 h at room temperature. After washing three times with TBS-Tween-20, nuclei were stained with propidium iodide, and finally the slides were mounted with fluoromount (Sigma, St. Louis, MO, USA) and observed under a microscope (Zeiss LSM-510, Oberkochen, Germany).

### 4.11. Statistical Analysis

For mean comparisons, we used analysis of variance (ANOVA). When the ANOVA test showed statistical differences, the Tukey post hoc test was used to discriminate between groups. Statistical significance was defined as *p* < 0.05 [79]. Statistical analyses were performed using GraphPad Prism version 5.0 for Windows (GraphPad Software, San Diego, CA, USA, www.graphpad.com).

## Supplementary Materials

Supplementary materials can be found at http://www.mdpi.com/1422-0067/19/8/2238/s1.
