# Supplementary material for: Bisphenol-A and Nonylphenol Induce Apoptosis in Reproductive Tract Cancer Cell Lines by the Activation of ADAM17
Source: Int J Mol Sci. 2018 Jul 31;19(8):2238. doi: 10.3390/ijms19082238 (PMC6121659; doi:10.3390/ijms19082238)
Supplement: Supplementary file 1 [file ijms-19-02238-s001.pdf]

Supplementary Materials

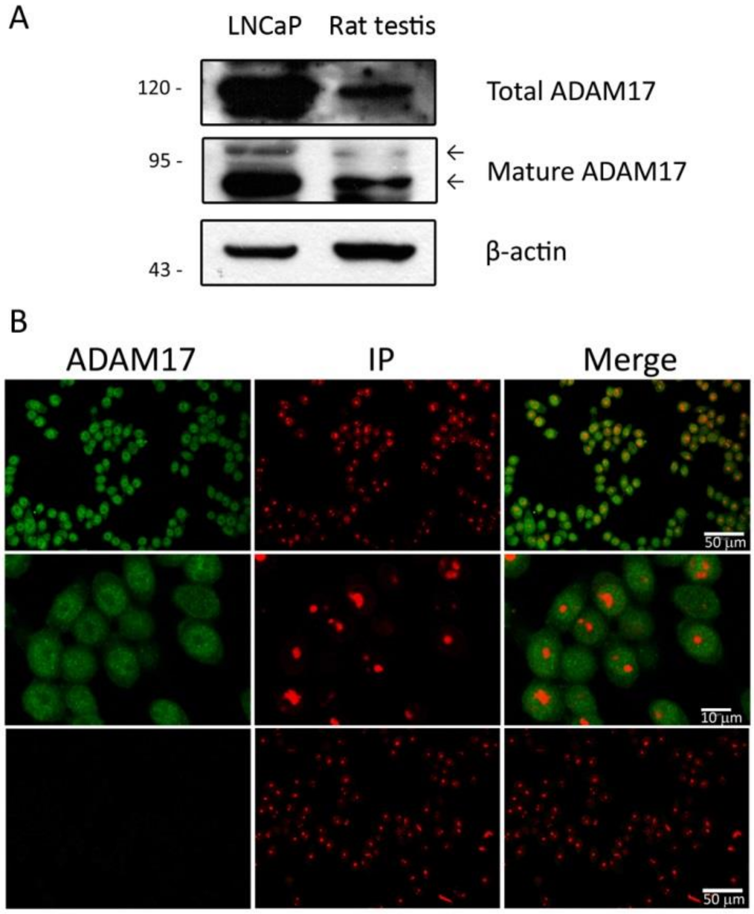

Figure S1. ADAM17 is expressed in LNCaP cell line. A) LNCaP cells and 21-day old rat testis (positive control) express immature and mature forms of ADAM17. β-actin was used as a loading control. B) The picture shows immunofluorescence against ADAM17 (green) in LNCaP cells; cell nuclei are in red stained with Propidium iodide (PI, red). Bars = 10 and 50 µm.

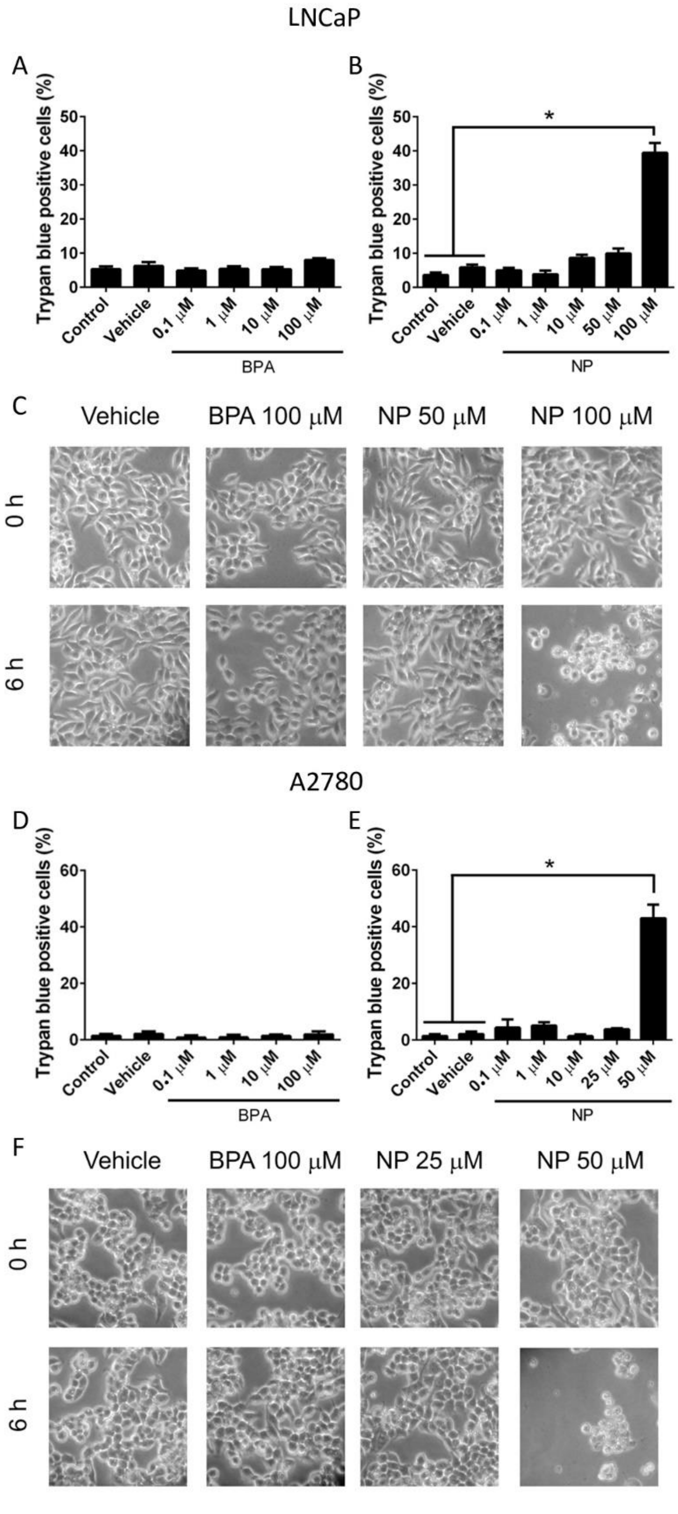

Figure S2. Trypan blue analysis after BPA and NP treatment. LNCaP cells were stimulated with different concentration of BPA (A) or NP (B) during 24 h. We observe an increase of Trypan blue positive cells with 100 μM NP but not with lower concentration or with BPA. (C) LNCaP contrast phase microscopy images at 0 h and 6 h of BPA and NP treatment. We observe changes in cellular morphology (cell death) only after treatment with 100 μM NP but not with 50 μM NP or 100 μM BPA. A2780 cells were stimulated with different concentration of BPA (D) or NP (E) during 2 h. We observe an increase of Trypan blue positive cells with 50 μM NP but not with lower concentration or with BPA. (F) A2780 contrast phase microscopy images at 0 h and 2 h of BPA and NP treatment. We observe changes in cellular morphology (cell death) only after treatment with 50 μM NP but not with 25 μM NP or 100 μM BPA. Vehicle: Ethanol, Mean ± SEM, * $p<0.05$, $n=3$.

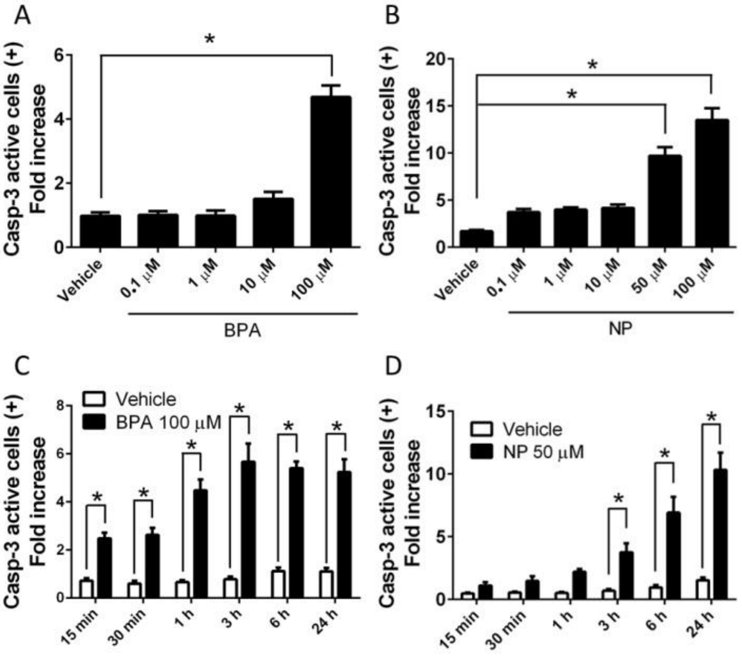

Figure S3. BPA and NP induce an increase of Caspase-3 active positive cells. Treatment with 100 μM BPA (A) or 50 and 100 μM NP (B) for 24 h induces a significant increase in the Caspase-3 active positive cell detected by immunohistochemistry. 100 μM BPA induces an increase in the Caspase-3 active positive cell from the 15 min of treatment (C), while 50 μM NP induces an increase from 3 h of treatment (D). Vehicle: Ethanol, Mean ± SEM, * p<0.05, n=3.

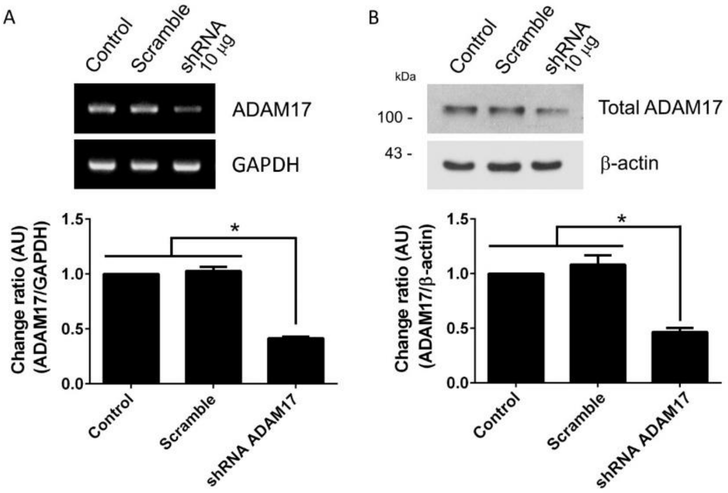

Figure S4. Transfection of A2780 cells with 10 μg of shRNA induces a robust decrease in the levels of ADAM17 mRNA (A) and protein (B). Mean ± SEM, * p<0.05, n=3.

Table S1: Phosphoproteins up- or down-regulated by 100 μM BPA or 50 μM NP in LNCaP cells after 15 min of treatment measured by a Human Phospho-Kinase Antibody Array.

Kinase	Phosphorylation Site	Change ratio (AU)		
		Control	BPA(100 μM)	NP(50 μM
p38	T180/Y182	1	2.45	0.71
ERK1/2	T202/Y204, T185/Y187	1	1.39	0.81
JNKpan	T183/185, T221/Y223	1	1.38	0.66
GSK-3α/β	S21/S9	1	1.75	0.66
p53	S392	1	1.01	1.01
EGF R	Y1086	1	2.83	1.15
MSK1/2	S376/S360	1	1.70	1.01
AMPKα1	T174	1	1.71	1.51
Akt	S473	1	2.14	1.21
Akt	T308	1	1.22	1.08
p53	S46	1	1.00	1.00
TOR	S2448	1	1.98	1.27
CREB	S133	1	3.05	0.95
HSP27	S78/S82	1	2.65	1.43
AMPKα2	T172	1	2.57	1.37
p70 S6 Kinase	T389	1	1.20	2.07
p53	S15	1	1.01	1.00
c-Jun	S63	1	2.22	2.84
Src	Y419	1	3.35	1.69
Lyn	Y397	1	2.56	0.99
Lck	Y394	1	3.48	1.41
STAT2	Y689	1	2.32	1.67
STAT5a	Y694	1	2.48	1.42
p70 S6 Kinase	T421/S424	1	1.39	1.23
RSK1/2/3	S380/S386/S377	1	1.17	1.17
eNOS	S1177	1	3.31	3.28
Fyn	Y420	1	3.37	1.65
Yes	Y426	1	2.97	1.07
Fgr	Y412	1	3.26	1.61
STAT6	Y641	1	2.54	1.62
STAT5b	Y699	1	2.86	1.51
STAT3	Y705	1	1.04	0.98
p27	T198	1	1.44	2.06
PLC-γ1	Y783	1	2.42	2.36
Hck	Y411	1	1.99	1.51
Chk-2	T68	1	3.79	1.40
FAK	Y397	1	2.42	1.16
PDGF Rβ	Y751	1	1.91	1.44

STAT5a/b	Y694/Y699	1	3.64	2.81
STAT3	S727	1	0.92	0.95
WNK1	T60	1	1.05	1.13
PYK2	Y402	1	6.01	8.62
PRAS40	T246	1	2.84	2.13
